# Body Mass Index and Caries: Machine Learning and Statistical Analytics of the Dental, Oral, Medical Epidemiological (DOME) Nationwide Big Data Study

**DOI:** 10.3390/metabo13010037

**Published:** 2022-12-26

**Authors:** Ofir Ben-Assuli, Ori Bar, Gaya Geva, Shlomit Siri, Dorit Tzur, Galit Almoznino

**Affiliations:** 1 Faculty of Business Administration, Ono Academic College, Kiryat Ono 55000, Israel; 2 Faculty of Dental Medicine, Hebrew University of Jerusalem, Jerusalem 91120, Israel; 3 Medical Information Department, General Surgeon Headquarter, Medical Corps, Israel Defense Forces, Tel-Hashomer, Ramat Gan 02149, Israel; 4 Faculty of Dental Medicine, Hebrew University of Jerusalem, Big Biomedical Data Research Laboratory, Dean’s Office, Hadassah Medical Center, Jerusalem, 91120, Israel; 5 Faculty of Dental Medicine, Hebrew University of Jerusalem, Department of Endodontics, Hadassah Medical Center, Jerusalem, 91120, Israel; 6 Faculty of Dental Medicine, Hebrew University of Jerusalem, Department of Oral Medicine, Sedation & Maxillofacial Imaging, Hadassah Medical Center, Jerusalem, 91120, Israel

**Keywords:** dental caries, decayed teeth, body mass index (BMI), obesity, Metabolic syndrome, data-driven analytics, big data, machine learning, algorithm, electronic medical record (EMR), electronic dental record (EDR)

## Abstract

The objectives of the research were to analyze the association between Body Mass Index (BMI) and dental caries using novel approaches of both statistical and machine learning (ML) models while adjusting for cardiovascular risk factors and metabolic syndrome (MetS) components, consequences, and related conditions. This research is a data-driven analysis of the Dental, Oral, Medical Epidemiological (DOME) big data repository, that integrates comprehensive socio-demographic, medical, and dental databases of a nationwide sample of dental attendees to military dental clinics for 1 year aged 18–50 years. Obesity categories were defined according to the World Health Organization (WHO): under-weight: BMI < 18.5 kg/m^2^, normal weight: BMI 18.5 to 24.9 kg/m^2^, overweight: BMI 25 to 29.9 kg/m^2^, and obesity: BMI ≥ 30 kg/m^2^. General linear models were used with the mean number of decayed teeth as the dependent variable across BMI categories, adjusted for (1) socio-demographics, (2) health-related habits, and (3) each of the diseases comprising the MetS definition MetS and long-term sequelae as well as associated illnesses, such as hypertension, diabetes, hyperlipidemia, cardiovascular disease, obstructive sleep apnea (OSA) and non-alcoholic fatty liver disease (NAFLD). After the statistical analysis, we run the XGBoost machine learning algorithm on the same set of clinical features to explore the features’ importance according to the dichotomous target variable of decayed teeth as well as the obesity category. The study included 66,790 subjects with a mean age of 22.8 ± 7.1. The mean BMI score was 24.2 ± 4.3 kg/m^2^. The distribution of BMI categories: underweight (3113 subjects, 4.7%), normal weight (38,924 subjects, 59.2%), overweight (16,966, 25.8%), and obesity (6736, 10.2%). Compared to normal weight (2.02 ± 2.79), the number of decayed teeth was statistically significantly higher in subjects with obesity [2.40 ± 3.00; OR = 1.46 (1.35–1.57)], underweight [2.36 ± 3.04; OR = 1.40 (1.26–1.56)] and overweight [2.08 ± 2.76, OR = 1.05 (1.01–1.11)]. Following adjustment, the associations persisted for obesity [OR = 1.56 (1.39–1.76)] and underweight [OR = 1.29 (1.16–1.45)], but not for overweight [OR = 1.11 (1.05–1.17)]. Features important according to the XGBoost model were socioeconomic status, teeth brushing, birth country, and sweetened beverage consumption, which are well-known risk factors of caries. Among those variables was also our main theory independent variable: BMI categories. We also performed clinical features importance based on XGBoost with obesity set as the target variable and received an AUC of 0.702, and accuracy of 0.896, which are considered excellent discrimination, and the major features that are increasing the risk of obesity there were: hypertension, NAFLD, SES, smoking, teeth brushing, age as well as our main theory dependent variable: caries as a dichotomized variable (Yes/no). The study demonstrates a positive association between underweight and obesity BMI categories and caries, independent of the socio-demographic, health-related practices, and other systemic conditions related to MetS that were studied. Better allocation of resources is recommended, focusing on populations underweight and obese in need of dental care.

## 1. Introduction

Standardized tests were designed to assess body fat, and a common method is to assess body mass index (BMI). The BMI is a simple to calculate tool, with high reliability, and correlation with both body fat and fat mass percentage [1]. BMI provides a more accurate assessment of total body fat than body weight alone [2]. In general, high BMI is a risk factor for developing chronic conditions such as diabetes, hypertension, depression, and cancer, and is typically used as a measurement to gauge the risk of developing these conditions [3]. Underweight BMI has been linked to a higher risk of illness and death (BMI ≤ 18.5 kg/m^2^) in Asia, Europe, and North America [4].

Populations of all ages are susceptible to caries, multifactorial bacterial disease of the oral cavity that is contagious, curable, and diet- and time-dependent [5]. It is a widespread illness that affects, up to 35% of the worldwide population, in all cultures, socio-economic statuses, sexes, and ethnicities [5]. Globally, in 2019, the prevalence was 2.03 billion (1.77 to 2.33) (46.07% increase), and 2.00 million (0.93–3.88) years lived with a disability (YLDs) (45.64% increase), all since 1990 [6]. Untreated caries continues to be a significant public health concern globally, yet demographic, sex, and regional patterns continue to vary [6].

Numerous research studies have investigated the connection between weight status and caries, particularly in children and adolescents, because health problems linked to growth and development and with oral disease may share a common pathway via dietary behaviors [7,8,9]. The main value proposition of linking dental data with BMI measurements is to identify populations at risk, so that health authorities could properly distribute resources and focus on tailoring them the required dental and medical care, according to evidence-based data.

However, the evidence in the literature of an association between BMI and caries was inconsistent [7,10], with some studies showing that increased BMI is associated with a higher burden of dental caries [11,12], other studies demonstrated that having a low weight is linked to having more caries [13,14] and other studies have not shown evidence that links these two variables [9,15]. The heterogeneity of results between studies could be attributed to different methods for caries assessment using only visual examination of decay compared to studies that used radiographs, lack of standardized cut-off points to classify BMI, and an absence or only partial adjustment for confounders and effect modifiers [7]. For instance, there are recognizable common risk factors for multiple chronic diseases, such as socioeconomic status, smoking, sugar consumption, and systemic co-morbidities [16]. A healthy diet including fruits and vegetables can supply the body with beneficial nutrients and antioxidants [17], including coenzyme Q10 and alpha-tocopherol, genistein proved to have a neuroprotective as they proved to have protective effects of antioxidants [18,19]. Silymarin is a herbal medicine with antioxidant and anti-inflammatory properties when given in patients with type 2 diabetes mellitus and has been proven to have superior efficacy compared with standard treatment alone [20]. Additionally, Daflon 500 mg (micronized purified flavonoid fraction of Rutaceae aurantiae, consisting of 90% diosmin and 10% hesperidin) proved to be helpful in reducing glucose levels and the risk of cardiovascular disease in type 2 diabetic patients [21].

Patient education is another factor important for the management of dental problems including dental caries which might be more significant than the therapy. Personal education about the procedures related to teeth brushing, the different types of toothbrush as well as the use of mouthwash. Periodontal disease is one of the two main and most prevalent oral diseases all over the world. Treatment strategies are diverse, where scaling and root planning (SRP) is the gold standard non-surgical therapy for periodontitis. Moreover, systemically administered antibiotics can be used as an adjunct to SRP to improve the treatment outcome of periodontitis. Abou El-Fadl et al. found that the adjunctive use of antibiotics had a significant effect on enhancing the clinical outcomes of therapy in chronic periodontitis patients and the clinical results for the patients who received patient education were more promising than those of patients who received periodontal treatment only [22].

Moreover, while most studies focused on children and adolescents, there is not much research examining the connection between adult BMI and dental health [12], in particular not in the age group of young to middle-aged adults. Among adults, the associations between caries and BMI should be assessed within the context of metabolic status while adjusting for cardiovascular risk factors. This is important since, in recent years, clinical phenotypes had been recognized of metabolically unhealthy normal weight and metabolically healthy obesity [23]. These phenotypes are not uncommon, and their increased risk of morbidity and mortality should not be overlooked [23,24]. When studying the association between BMI and caries, the parameters of metabolic morbidity were even less considered in the literature compared to other effect modifiers, since most studies were conducted in children and adolescents.

Considering this gap in the literature, there is a need to perform large-scale epidemiological research on the association between BMI and dental caries that employs a rigorous protocol regarding BMI cut-off definitions as well as caries assessment including mandatory radiographs in addition to visual inspection and considering the existence of many possible confounders and effect modifiers such as socio-demographics, health-related habits, and metabolic morbidities. While most studies used only statistical models to address the subject, recently, machine learning (ML) approaches in artificial intelligence were used to select the most relevant variables (aka feature selection/feature importance) in identifying root caries [25] and early childhood caries [26] using various machine learning as support vector machine, XGBoost and Random Forest [25], Light Gradient Boosted Machine, logistic regression (including regression-based backward elimination) [26]. To the best of our knowledge employment of statistical as well as ML models, in the context of BMI categories, cardiometabolic risk factors, and dental caries were not published yet in the English literature. Therefore, the main contributions of this work are the exploration of the association between BMI and caries using a large-scale, structured and comprehensive database among a nationwide representative sample and the use of novel statistical and ML approaches, which have not been carried out before.

To address this literature gap, the main goal of this research was to study the association between BMI and caries in an Israeli nationwide representative sample of young and middle-aged adults. The null hypothesis (H0) of this study was that there is no association between BMI and the number of decayed teeth. The alternative hypothesis (H1) was that lower BMI measurement (underweight) and higher BMI categories (obesity) are both associated with more decayed teeth, even after controlling for potential confounding and effect-modifying factors. To that end, the specific research goals were:

1. To explore the associations of decayed teeth as a dependent variable with different BMI categories in various statistical models adjusted for potential confounding factors, such as (1) socio-demographic variables: age, sex, educational level, socio-economic status (SES), residency, and country of birth; and (2) health-related habits: smoking, teeth brushing, cariogenic nutrition, and sugary drinks as well as other diseases comprising the Metabolic Syndrome (MetS) including, hypertension, diabetes, hyperlipidemia, cardiovascular disease, nonalcoholic Fatty Liver Disease (NAFLD), and obstructive sleep apnea (OSA).
2. To employ supervised machine learning (ML) algorithms that will explore the relative clinical features importance for two targets: (a) the dichotomous variable of decayed teeth and (b) obesity (BMI ≥ 30 kg/m^2^), while using the same set of clinical features that were used in the statistical models.
3. To compare the results obtained by the statistical and ML models and discuss and summarize the conclusions.

## 2. Methods

### 2.1. Data Source

This study is a records-based cross-sectional study. The study is a part of the dental, oral, and medical epidemiological (DOME) study [27,28,29,30,31]. Complete information on the methods of the DOME has been depicted in our previous publication [27]. The DOME is a structured comprehensive study based on big data, including the demographics, dental, and medical profiles of military personnel ranging from young to middle-aged [27,28,29,30,31]. Thus, the DOME is a powerful tool that provides an exceptional opportunity to cross-reference dental outcomes with BMI measurements to investigate the associations between BMI categories and caries, whilst also adjusting for socio-demographics, health-associated practices, and systemic conditions related to metabolic syndrome. It should be noted that in Israel, service in the military is compulsory by law for every qualified Jewish, Druze, or Circassian citizen older than 18, and consequently, the Israeli military population is large and provides a solid source of data for population-based epidemiologic studies in young and middle-aged adults [27]. Of importance is the fact that the service in the IDF includes individuals with a complex medical background, excluding subjects who are unqualified to serve because of significant health problems (physically or mentally), and even exempted recipients have the option of applying for volunteer service [27,32]. Dental care is included in the extensive healthcare basket and is provided to military members free of charge [27]. The Department of Medical Information of the Medical Corps carried out data mining, and the dataset is anonymous [27].

### 2.2. Ethical Approval

Approval to perform the study was given by the Institutional Review Board of the IDF Medical Corps (protocol code: IDF-1281-2013). Considering that the study was retrospective, including anonymous records analyses, an exemption from written informed consent was given by the IRB.

### 2.3. Data Collection

Our previous publication contains a thorough explanation of the data collection of the DOME research [27]. Briefly, the DOME structured repository captures 3 military electronic databases: Dental Patient Record (DPR), medical (i.e., computerized patient record (CPR)), and socio-demographic computerized systems that store personal [27] socio-demographic profiles and dental and medical records of all military personnel.

### 2.4. Eligibility Criteria

**Inclusion criteria:** The socio-demographics, health, and dental data of military members of the IDF, men, and women aged 18 years and older, who visited the IDF Dental Corps clinics between 1 January 2015 and 1 January 2016, and for whom records exist in the socio-demographic medical, and dental military records.

**Exclusion criteria:** Subjects with a lack of data in these databases.

### 2.5. Definition of Variables

**Dental Caries**. The number of decayed teeth was derived from the dental patients’ records. The standard processes of administration and clinical workup, as well as quality assurance (QA) applied by the Dental Corps, are detailed within the DOME protocol publication [27]. In summary, the DOME database captures standardized dental codes that correspond to the definitions in use by the American Dental Association’s (ADA) current dental terminology (CDT). All dental patients had indoor dental evaluations, which included bi-lateral bite wings molar and premolar regions, as well as a periapical X-ray to assess deep dental cavities, teeth with endodontic treatment, and periodontitis. [27].

**Body Mass Index (BMI)**. Was retrieved from the CPR [27]. BMI was computed as weight in kilograms divided by height in meters squared, which had been calculated from measured weight and height (bare feet and in underwear) measured by qualified medical personnel that uses a beam balance and stadiometer [27,33]. Physical examinations of included measurements of weight and height are routinely documented and weight is rounded to the closest 0.5 kg, while height is to the closest and 1 cm [27,33]. We utilized the world health organization (WHO) adult BMI categories [34], and for analysis, the BMI scores were divided into classes according to the WHO: underweight (BMI of less than 18.5), normal weight (BMI of > 18.5 to 25), overweight (BMI > 25 to 30), and obesity (BMI of more than 30) [34].

**Socio-Demographics and health-related practice parameters**. The DOME protocol publication contains extensive details on the socio-demographic and health-related practices definitions [27]. Socio-demographic variables that were included appear in Table 1, and health-related practices in Table 2, both Tables are in the Section 3.

**Definitions of Medical Diagnoses**. The medical diagnosis diseases comprising the MetS definition, MetS, and long-term sequelae as well as associated illnesses, were drawn from the medical records and were based on the ICD-9-CM, as described previously [21,22,25]. Medical diagnoses included hypertension, diabetes, hyperlipidemia, non-alcoholic, nonalcoholic Fatty Liver Disease (NAFLD), obstructive sleep apnea (OSA), and cardiovascular disease.

### 2.6. Data Analysis

**Scheme 1:**
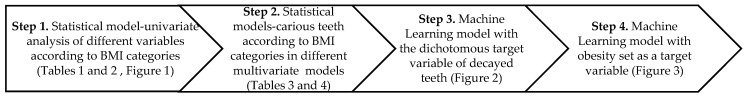
Flow chart of data analyses approach.

The approach used to analyze the data is illustrated in Scheme 1 and described in detail in Section 2.6.1 and Section 2.6.2.

#### 2.6.1. Statistical Analysis

Following data tabulation, the statistical analyzes were conducted utilizing Statistical Package for the Social Sciences (SPSS) software version 27.0 International Business Machines (IBM), Chicago, IL, USA.

Average and standard deviation are used to display continuous parameters, and absolute numbers and percentages are used to represent categorical parameters.

Step 1: BMI analysis was a categorical variable comprised of 4 categories: underweight, normal weight, overweight, and obesity. Statistical tests used to analyze the socio-demographics, health-related practices, systemic conditions and the mean number of decayed teeth across BMI categories were Analysis of variance (ANOVA) (for continuous parameters) and Likelihood ratio (for categorical variables). Continuous variables did not distribute normally. Nevertheless, since the results of the nonparametric Kruskal–Wallis test was similar to the ANOVA, and since the sample size was large, ANOVA results are displayed. Since the sample size was large, *p* < 0.01 (2-tailed) was deemed statistically significant.

Step 2: Several general linear regressions models (GLM) were utilized to measure the odds ratios (OR) and 95% confidence intervals (CI) for the dependent variable, i.e., the mean number of decayed teeth according to the 4 categories of BMI, controlling for sociodemographic, health-related practices, and medical diagnoses. The following models were used to study decayed teeth–BMI association:

1st Model without adjustment; 2nd model: adjustment for age; 3rd model: adjustment included age and sex; 4th model: 3rd model parameters with educational level; 5th model: 4th model parameters with socioeconomic status (SES); 6th model: 5th model parameters with residence location; 7th model: 6th model parameters with birth countries; 8th model: 7th model parameters with hypertension; 9th model: 8th model parameters with diabetes mellitus; 10th model: 9th model parameters with hyperlipidemia; 11th model: 10th model parameters with nonalcoholic Fatty Liver Disease (NAFLD), 12th model: 11th model with obstructive sleep apnea (OSA); 13th model: 12th model with cardiovascular disease; 14th model: 13th model parameters with smoking; 15th model: 14th model parameters and tooth brushing; 16th model: 15th model parameters with cariogenic nutrition and sugary drinks. The last 16th model is displayed with collinearity statistics. Variance inflation factors (VIF), equal to 1 ÷ Tolerance were calculated using linear regression. Even though VIF above 10 is deemed as implying collinearity, a problem could occur in weakened models if the VIF is over 3.5, and hence, we set the VIF threshold at 3.5.

#### 2.6.2. Sub-Section Clinical Features Importance Based on Machine Learning Algorithms

To explore the relative clinical features’ importance of the targets we used XGBoost [35], which serves as an efficient gradient-boosting framework used for supervised machine learning for both regression and classification problems. We explored the relative clinical features importance of two targets: (a) the dichotomous variable of decayed teeth (step 3) and (b) obesity (BMI ≥ 30 kg/m^2^) (step 4) while using the same set of clinical features that were used in the statistical models. All models were implemented using python using the scikit-learn package [36]. We have run the model with various proportions of Training and Testing datasets (e.g., Train-Test: 70–30% and 80–20%), with five-fold cross-validation.

Sensitivity analyses: to validate the stability of the XGBoost model, we also run two additional selected methods for feature importance: Gini Importance [37] and Information Gain (using Entropy) [38], and received quite similar goodness-of-fit model measurements [e.g., area under the curve (AUC) and accuracy].

## 3. Results

### 3.1. Socio-Demographics across BMI Categories

The study included 65,739 subjects with a mean age of 22.8 ± 7.1. The mean BMI score was 24.2 ± 4.3 kg/m^2^, median 23.58, mode 22.86, and range of 13.76–47.83 kg/m^2^. Table 1 presents the socio-demographics of the study population across the four BMI categories. The purpose of the tests presented in Table 1 is to control BMI categories for socio-demographic parameters (univariate analysis). BMI categories distributed as follows among the study population: underweight (3113 individuals, 4.7%), normal weight (38,924, 59.2%), overweight (16,966, 25.8%) and obesity (6736, 10.2%) (Table 1). Age, higher education, and low SES were positively associated with BMI categories with a dose–response curve from the lower to higher BMI category. There was a higher proportion of women, high school education, urban Jewish locality, and birth country from East Europe and Ethiopia in the underweight category, compared to other BMI categories (see Table 1).

**Table 1:** Socio-demographics of the study population across the four Body Mass Index (BMI) categories * ANOVA, ˅ Likelihood ratio, SES: Socio-economic status, FSU: Former Soviet Union.

**Parameter**		**BMI Categories**				**Total (%) or Mean ± SD**	***p* Value**
		**Underweight**	**Normal Weight**	**Overweight**	**Obesity**		
Number (%)		3113 (4.7)	38,924 (59.2)	16,966 (25.8)	6736 (10.2)	65,739 (100)	
Age (years)		19.9 ± 3.2	21.5 ± 5.7	25.2 ± 8.5	26.4 ± 9.0	22.8 ± 7.1	<0.001 *
Sex	Men	1556 (50.0)	28,397 (73.0)	14,113 (83.2)	5342 (79.3)	49,408 (75.2)	<0.001 ˅
	Woman	1557 (50.0)	10,527 (27.0)	2853 (16.8)	1394 (20.7)	16,331 (24.8)	
Education	High school	2910 (93.7)	33,064 (85.1)	11,716 (69.1)	4417 (65.7)	52,107 (79.4)	<0.001 ˅
	Technician	75 (2.4)	1865 (4.8)	1940 (11.4)	1038 (15.4)	4918 (7.5)	
	Academics	120 (3.9)	3937 (10.1)	3289 (19.4)	1272 (18.9)	8618 (13.1)	
SES	Low	128 (4.1)	1642 (4.3)	951 (5.7)	4141 (6.3)	3135 (4.8)	<0.001 ˅
	Medium	1654 (53.5)	19,419 (50.5)	9071 (54.5)	3917 (59.2)	34,061 (52.6)	
	High	1307 (42.3)	17,364 (45.2)	6636 (39.8)	2288 (34.6)	27,595 (42.6)	
Locality of residence	Urban Jewish	2828 (91.2)	33,151 (85.6)	14,276 (84.7)	5831 (87.1)	56,086 (85.8)	<0.001 ˅
	Urban non-Jewish	267 (8.6)	5369 (13.9)	2401 (14.2)	792 (11.8)	8829 (13.5)	
	Rural	5 (0.2)	196 (0.5)	181 (1.1)	75 (1.1)	557 (0.7)	
Birth Country	Western Europe	38 (1.2)	896 (2.3)	526 (3.1)	223 (3.3)	1683 (2.6)	<0.001 ˅
	Eastern Europe	238 (7.7)	2196 (5.6)	982 (5.8)	412 (6.1)	3828 (5.8)	
	FSU	42 (1.4)	450 (1.2)	207 (1.2)	102 (1.5)	801 (1.2)	
	Asia	3 (0.1)	73 (0.2)	64 (0.4)	23 (0.3)	163 (0.2)	
	East Asia	5 (0.2)	57 (0.1)	25 (0.1)	7 (0.1)	94 (0.1)	
	Ethiopia	129 (4.1)	832 (2.1)	152 (0.9)	22 (0.3)	1135 (1.7)	
	Africa	5 (0.2)	99 (0.3)	75 (0.4)	29 (0.4)	208 (0.3)	
	North America	39 (1.3)	995 (2.6)	441 (2.6)	112 (1.7)	1587 (2.4)	
	South America	8 (0.3)	298 (0.8)	168 (1.0)	60 (0.9)	534 (0.8)	
	Oceania	1 (0.0)	47 (0.1)	14 (0.1)	3 (0.0)	65 (0.1)	
	Israel	2603 (83.7)	32,974 (84.7)	14,306 (84.4)	5741 (85.3)	55,624 (84.6)	

### 3.2. Mean Number of Decayed Teeth across BMI Categories

**Figure 1:**
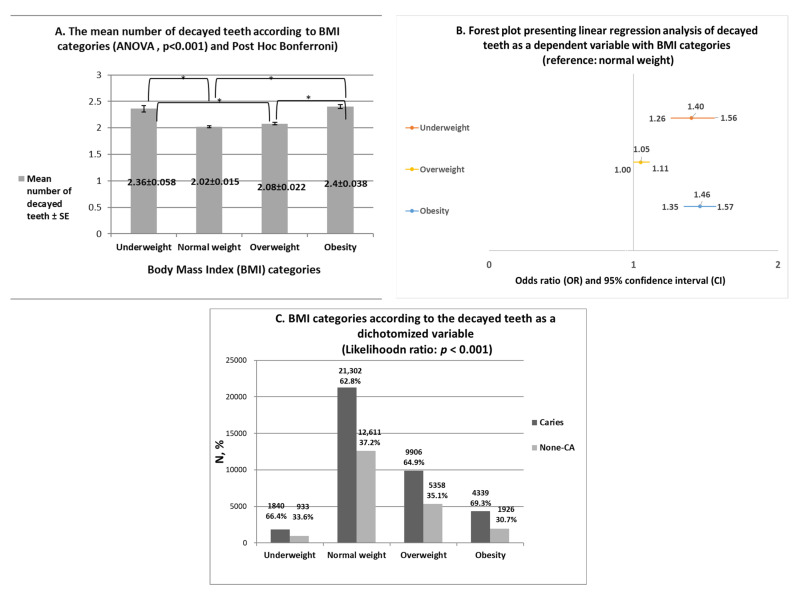
(**A**). ANOVA analysis of the mean number of decayed teeth according to BMI categories:The mean number of decayed teeth according to the four BMI categories: *p* < 0.001, followed by Post hoc Bonferroni tests: Underweight vs. normal weight (*p* < 0.001) and overweight (*p* < 0.001), Normal weight vs. obesity (*p* < 0.001) Overweight vs. obesity (*p* < 0.001). * Statistically significant values in the Post hoc Bonferroni tests. There were no statistically significant differences between normal weight and overweight. (**B**). Forest plot presenting linear regression analysis of the mean number of decayed teeth as a dependent variable with BMI categories (reference: normal weight). Results show a statistically significant positive association with underweight (OR = 1.40 (1.26–1.56)], and obesity [OR = 1.46 (1.35–1.57), and ORs close to 1 in the overweight category [OR = 1.05 (1.00–1.11). (**C**). The mean number of decayed teeth as a dichotomized variable [caries and None-CA (caries)] across BMI categories using Likelihood ratio test. Results show higher proportion of subjects with caries in the underweight (66.4%) and obesity (69.3%) categories compared to the normal weight (62.8%) and overweight (64.9%) categories (*p* < 0.001).

The purpose of the tests presented in Figure 1 is to perform univariate analyses to study the associations between BMI categories and caries Figure 1A presents an analysis of the mean number of decayed teeth of the study population across BMI categories. The average number of decayed teeth was greater in the underweight (mean ± standard error: 2.36 ± 0.058) and obesity (2.40 ± 0.038) categories compared to normal weight (2.02 ± 0.015) and overweight (2.08 ± 0.022) (*p* < 0.001, Figure 1A).

Figure 1B presents a forest plot presenting linear regression analysis of the mean number of decayed teeth as a dependent variable with BMI categories (reference: normal weight). As can be seen in Figure 1B, decayed teeth had a statistically significant positive association with underweight [Odds ratio (OR) and 95% confidence interval (CI) = 1.40 (1.26–1.56)], and obesity [OR = 1.46 (1.35–1.57)] compared to normal weight (reference) (Figure 1B). ORs were close to 1 in the overweight category compared to the normal weight [OR = 1.05 (1.00–1.11), *p* < 0.001] (Figure 1B).

In the next step (Figure 1C), analysis of the main endpoint of the current research, i.e., the mean number of decayed teeth, as a dichotomized variable as follows: (1) Caries = the existence of one decayed tooth or more in the dental assessment; (2) None-caries (none-CA) = absence of any decayed tooth in the dental assessment. As can be seen in Figure 1C, there was a higher proportion of subjects with caries in the underweight (66.4%) and obesity (69.3%) categories compared to the normal weight (62.8%) and overweight (64.9%) categories (likelihood ratio: *p* < 0.001).

### 3.3. Health-Related Practices and Medical Diagnoses Related to Metabolic Syndrome (MetS) across BMI Categories

Table 2 presents the health-related practices and medical diagnoses related to MetS of the study population across the four BMI categories. The purpose of the tests presented in Table 2 is to control BMI categories for health-related practices and systemic conditions related to MetS (univariate analysis). Smoking, brushing teeth less than daily, the consumption of cariogenic nutrition and sugary drinks, and all systemic conditions related to MetS (hypertension, diabetes, hyperlipidemia, NAFLD, OSA, and cardiovascular disease) were positively associated with BMI categories with a dose–response curve from lower to higher BMI category (Table 2).

**Table 2:** Health-related practices and metabolic morbidity of the study population across the four Body Mass Index (BMI) categories, ˅ Likelihood ratio, FSU: Former Soviet Union.

**Parameter**		**BMI Categories**				**Total (%) or Mean ± SD**	***p* Value**
		**Underweight**	**Normal Weight**	**Overweight**	**Obesity**		
Number (%)		3113 (4.7)	38,924 (59.2)	16,966 (25.8)	6736 (10.2)	66,790 (100)	
Smoking	No	3033 (97.4)	37,210 (95.6)	14,854 (87.6)	5541 (82.3)	60,638 (92.2)	<0.001 ˅
	Yes	80 (2.6)	1714 (4.4)	2112 (12.4)	1195 (17.7)	5101 (7.8)	
Brushing teeth at least once a day	No	108 (10.2)	1262 (10.6)	607 (12.2)	359 (17.7)	2336 (11.7)	<0.001 ˅
	Yes	946 (89.8)	10,700 (89.4)	4365 (87.8)	1167 (82.3)	17,678 (88.3)	
Consumption of cariogenic nutrition	No	440 (41. 8)	5879 (49.2)	2602 (52.4)	1016 (50.3)	9937 (49.7)	<0.001 ˅
	Yes	613 (58.2)	6074 (50.8)	2366 (47.6)	1004 (49.7)	10,057 (50.3)	
Consumption of sugary drinks	No	452 (43.0)	5509 (46.1)	2346 (47.3)	887 (43.9)	9194 (46.0)	0.014 ˅
	Yes	598 (57.0)	6429 (53.9)	2167 (52.7)	1133 (56.1)	10,777 (54.0)	
Hypertension	No	3090 (99.3)	38,283 (98.4)	16,034 (94.5)	5813 (86.3)	63,220 (96.2)	<0.001 ˅
	Yes	23 (0.7)	641 (1.6)	932 (5.5)	923 (13.7)	2519 (3.8)	
Diabetes	No	3111 (99.9)	38,865 (99.8)	16,854 (99.3)	6614 (98.2)	65,444 (99.6)	<0.001 ˅
	Yes	2 (0.1)	59 (0.2)	112 (0.7)	122 (1.8)	295 (0.4)	
Hyperlipidemia	No	3110 (99.9)	38,704 (99.4)	16,614 (97.9)	6555 (97.3)	64,983 (98.8)	<0.001 ˅
	Yes	3 (0.1)	220 (0.6)	352 (2.1)	181 (2.7)	756 (1.2)	
Non-alcoholic fatty liver disease (NAFLD)	No	3112 (100)	38,837 (9.8)	16,657 (98.2)	6338 (94.1)	64,944 (98.8)	<0.001 ˅
	Yes	0 (0)	87 (0.2)	309 (1.8)	398 (5.9)	795 (1.2)	
Obstructive sleep apnea (OSA)	No	3113 (100)	38,878 (99.9)	16,867 (99.4)	6638 (98.5)	65,496 (99.6)	<0.001 ˅
	Yes	0 (0)	46 (0.1)	99 (0.6)	98 (1.5)	243 (0.4)	
Cardiovascular disease	No	3038 (97.6)	37,919 (97.4)	16,162 (95.3)	6297 (93.5)	63,416 (96.5)	<0.001 ˅
	Yes	75 (2.4)	1005 (2.6)	804 (4.7)	439 (6.5)	2323 (3.5)	

### 3.4. Carious Teeth According to BMI Categories in Different Multivariate Analyses Models

Table 3 presents the analysis of carious teeth (continuous variable) according to BMI categories in various general linear regression models (GLM), with adjustment for possible confounders and independent risk factors that include socio-demographic parameters, health-related practices and systemic conditions related to MetS In all models, the normal weight category was set as a reference. In the first unadjusted model (model 1, see also Figure 1B), there was a 1.40-fold increase in the OR for decayed teeth in the underweight category [OR = 1.40 (1.26–1.56), *p* <0.001], and 1.46-fold higher in the obesity category [OR = 1.46 (1.35–1.57), *p* <0.001], compared with the normal weight. ORs were close to 1 in the overweight category compared to the normal weight [OR = 1.05 (1.00–1.11), *p* < 0.001]. Further adjustments were performed for socio-demographic parameters (models number 2 to 7), parameters associated with systemic morbidity related to (models 8–13), and parameters associated with health-related practices (models 14–16) (see Table 3). Across the models, the statistically significant ORs were not eliminated for underweight and even became higher for obesity, but overweight lost statistical significance with decayed teeth in the last model (Table 3).

Table 4 displays the final GLM including the multicollinearity statistics (16th model). The purpose of this model is to show the parameters that retain a statistically significant association after multivariate analysis that controlled for the maximal number of confounders including socio-demographics, health-related habits and systemic conditions. Table 4 shows that multicollinearity was excluded (VIF < 3.5). The parameters that maintained their statistically significant positive association with carious teeth in the final 16th model were (from maximal to minimal OR): birth country Eastern Europe vs. native Israeli [OR = 3.33 (2.49–4.45)], consumption of sugary drinks [OR = 1.65 (1.50–1.81)], obesity [OR = 1.56 (1.39–1.76)], underweight [OR = 1.29 (1.16–1.45)], smoking [OR = 1.26 (1.11–1.44)], male sex [OR = 1.16 (1.07–1.26)], consumption of cariogenic nutrition [OR = 1.16 (1.06–1.27)].

Decayed teeth retained a statistically significant negative association with age [OR = 0.989 (0.981–0.997)], birth country North America vs. native Israeli [OR = 0.53 (0.40–0.69)], brushing teeth at least once a day [OR = 0.53 (0.49–0.58)],

Decayed teeth did not retain a statistically significant association with overweight [OR = 1.04 (0.96–1.13)] and with the systemic conditions related to MetS (except BMI categories) including hypertension [(OR = 1.08 (0.91–1.29)], diabetes [OR = 1.25 (0.81–1.93)], hyperlipidemia [OR = 0.86 (0.65–1.16)], NAFLD [OR = 0.89 (0.67–1.17)], OSA [OR = 0.69 (0.43–1.09)] and cardiovascular disease [OR = 1.07 (0.90–1.28)] (Table 4).

### 3.5. Clinical Features Importance Based on Machine Learning Algorithms

We run XGBoost on the same set of clinical features that were used in the statistical models to explore the relative clinical features’ importance based on an advanced ML algorithm.

#### 3.5.1. Clinical Features Importance Based on XGBoost Machine Learning Model with the Dichotomous Target Variable of Decayed Teeth

**Figure 2:**
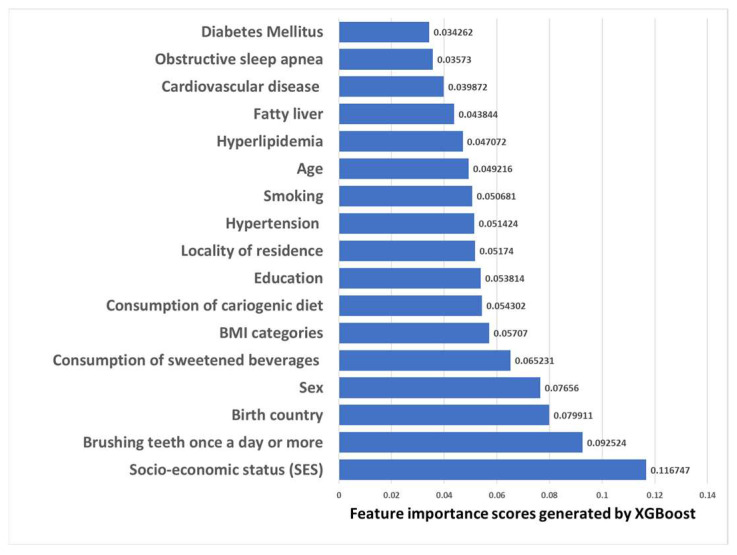
Clinical Features Importance ranking chart based on XGBoost Machine Learning algorithm for the dichotomous decayed teeth set as target variable. Values are the importance rankings of the features ranks from highest (SES) to lowest (Diabetes Mellitus), with BMI categories ranked fifth in features importance.

The purpose of the XGBoost model shown in Figure 2 is to find the important features to predict dental caries. Overall, on a threshold value of 0.65 (like the general population average of caries in this study, see Figure 2), we received an AUC of 0.602 and an accuracy of 0.66. The AUC of this model is considered acceptable discrimination. The results in Figure 2 illustrate that among the major features that are increasing the risk of caries there are: SES, teeth brushing, birth country, consumption of sweetened beverages, as well as our main theory independent variable: BMI categories.

#### 3.5.2. Clinical Features Importance Based on XGBoost Machine Learning Model with Obesity Set as a Target Variable

**Figure 3:**
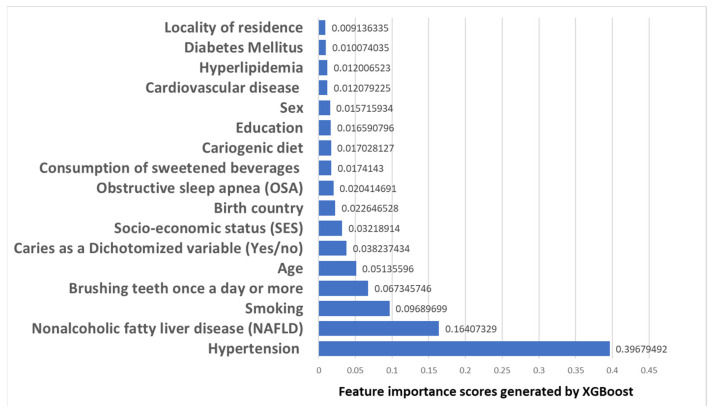
Clinical Features Importance ranking chart based on XGBoost Machine Learning algorithm for obesity (BMI ≤ 30 kg/m^2^) set as target variable. Values are the importance rankings of the features ranks from highest (hypertension) to lowest (locality of residence), with caries as a dichotomized variable ranked sixth in features importance.

The purpose of the XGBoost model shown in Figure 3 is to find the important features to predict obesity. The AUC was 0.702, and the accuracy of 0.896, which is considered excellent discrimination. The results in Figure 3 illustrate that among the major features that are increasing the risk of obesity there are: hypertension, NAFLD, SES, smoking, teeth brushing, age as well as our main theory dependent variable: caries as a Dichotomized variable (Yes/no).

## 4. Discussion

The present study demonstrated that being underweight and obese was positively associated with a higher mean number of decayed teeth. To investigate the association between BMI categories and the dependent variable carious teeth we utilized various models. In the current research, the associations between BMI categories and carious teeth remained even following adjustment for numerous confounding and common risk factors for caries, supporting our hypothesis that there is an independent association between underweight and obesity and caries. Included parameters were socio-demographics (age, sex, educational level, SES, residence location, and birth countries) and health-related practices (smoking, tooth brushing, cariogenic nutrition, and sugary drinks) as well as systemic conditions related to MetS (hypertension, diabetes, hyperlipidemia, NAFLD, OSA, and cardiovascular disease) To support the statistical models, we used XGboost ML algorithm using the variables that were employed in the statistical model. The results in Figure 2 illustrate that among the major features that are increasing the risk of caries there are not only well-known risk factors for caries: SES, teeth brushing, birth country, consumption of sugary drinks but also BMI categories, which reached the fifth place in the model. The AUC of this model is considered acceptable discrimination. We further run another XGboost with obesity set as the target, and the feature selected by the algorithm to be the major features that are increasing the risk of obesity were hypertension, NAFLD, SES, smoking, age as well as teeth brushing (reached third place) and our main theory dependent variable: caries as a dichotomized variable (reached fifth place). This model received an AUC which is considered excellent discrimination. Overall, the results support the study hypothesis that lower BMI scores (underweight), and higher BMI categories (obesity) both have a positive association with caries, despite adjustment for possible confounders and effect modifiers. The current research utilized for the analyses of BMI and caries associations the large nationwide sample of young to middle-aged adults. To the best of our knowledge, this has been the first study to perform detailed analyses that crossed dental caries with BMI categories and used novel approaches of both statistical and ML models among a nationwide sample including comprehensive data of socio-demographics, health-related practices, and systemic conditions.

There are potential explanations for the positive association of underweight and obesity with carious teeth. Initially, due to common socio-demographic risk factors for underweight, obesity, and dental caries such as education and SES, the observed associations may reflect these already known associations between education, SES, dental caries, and BMI. In the literature, SES was found to correlate negatively with obesity [39], and with caries [40], and it was suggested that obesity be viewed as a social phenomenon, containing both economical and sociocultural elements such as maternal education and self-esteem [41]. However, the association of underweight and obesity with carious teeth was retained following adjusting for the socio-demographics variables, and therefore makes this explanation less probable, although it cannot be ruled out. Indeed, low SES retained a statistically significant positive association with a higher mean number of decayed teeth in the final multivariate model 16 (see Table 4) and was also located as the first feature selected by the ML algorithm for dichotomized decayed teeth as a target (see Figure 2), and as the third feature selected by the ML algorithm for obesity as a target (see Figure 3).

A further possibility is that the observed caries variability in different BMI categories is due to different health-related practices. Caries is linked to unhealthy lifestyle habits such as smoking, inadequate teeth brushing, and sugary foods [42]. Higher A behavioral explanation stands, as heavy smokers are more prone to behave in a weight-gaining manner (for instance low physical activity, bad diet, alcohol intake) compared to others [43]. However, again, since being underweight and obese retained their positive association with carious teeth despite adjusting for health-related lifestyle habits variables, and therefore this explanation is less probable, although it cannot be ruled out.

The present study considered not only socio-demographic parameters and health-related habits but also adjusted for systemic conditions associated with MetS. This is important to discriminate between metabolically unhealthy normal weight and metabolically healthy obesity. None of the systemic conditions related to MetS except BMI categories retained a statistically significant association with decayed teeth following multivariate analysis (model 16, Table 4), and systemic conditions were also located downward compared to BMI categories in feature selection for decayed teeth as target (Figure 2). This makes it less likely that systemic conditions are the sole expansion for the association between BMI and dental caries, even when considering obesity-related conditions such as OSA and possible “diabesity” profiles.

### Strength and Limitations

A large number of subjects (66,790 subjects) of a nationally representative sample of young and middle-aged adults, as well as the usage of the DOME database that encompasses BMI testing measurements, socio-demographics, dental, lifestyle habits, and MetS-related systemic morbidities, are the main strength of the study. As Israel is an immigrant country, the study included a variety of ethnic groups, allowing for reference with other populations. Standardized definitions were used for all people, and all parameters analyzed were validated in prior studies. We also used both statistical and ML models to study the associations between BMI and caries. Limitations include the fact that while numerous parameters were taken into consideration since the topic is complex, other variables were not examined. These include parents’ history, childhood, and in utero exposures, genetics, previous lifestyle practices and history of medication, teeth health and BMI. Furthermore, because this research was cross-sectional, causality cannot be assumed, and thus only associations between the parameters are discussed. As the participants in this research were military personnel, the findings of the current research might not be generalizable to the general population. 

## 5. Conclusions

This research demonstrates a positive association between underweight and obesity BMI categories and dental caries, independent of the socio-demographic, health-related practices, and other systemic conditions related to MetS that were studied. Better resource distribution is suggested, which will focus on underweight and obese populations who require dental treatment. It is suggested to conduct future longitudinal studies including genetics and epidemiological data to uncover the origins and pathways behind the findings of this research.

## Figures and Tables

**Table 3:** Odds ratios (ORs) for carious teeth according to BMI categories in different general linear regression models (GLM). Description of the model applied is in background color.

Variable	BMI Categories			
	Normal Weight	Underweight	Overweight	Obesity
**1st Model without adjustment: carious teeth across BMI categories**				
OR and 95%CI	1	1.40 (1.26–1.56)	1.05 (1.00–1.11)	1.46 (1.35–1.57)
**2nd Model: 1st Model adjusted for age**				
OR and 95%CI	1	1.33 (1.19–1.48)	1.19 (1.12–1.26)	1.70 (1.58–1.84)
**3rd Model: 2nd model parameters with sex**				
OR and 95%CI	1	1.42 (1.27–1.58)	1.16 (1.09–1.22)	1.68 (1.56–1.82)
**4th Model: 3rd model parameters with educational level**				
OR and 95%CI	1	1.39 (1.24–1.55)	1.15 (1.09–1.21)	1.63 (1.50–1.76)
**5th Model: 4th model parameters with socio-economic status (SES)**				
OR and 95%CI	1	1.35 (1.21–1.50)	1.10 (1.05–1.17)	1.53 (1.41–1.65)
**6th model: 5th model 5 parameters with residence location**				
OR and 95%CI	1	1.32 (1.18–1.47)	1.11 (1.05–1.17)	1.51 (1.40–1.64)
**7th model: 6th model parameters with birth countries**				
OR and 95%CI	1	1.29 (1.16–1.44)	1.11 (1.05–1.17)	1.51 (1.40–1.64)
**8th model: 7th model parameters with hypertension**				
OR and 95%CI	1	1.29 (1.16–1.44)	1.11 (1.05–1.17)	1.51 (1.39–1.63)
**9th model: 8th model parameters with diabetes mellitus**				
OR and 95%CI	1	1.29 (1.16–1.44)	1.11 (1.05–1.17)	1.51 (1.39–1.63)
**10th model: 9th model parameters with hyperlipidemia**				
OR and 95%CI	1	1.29 (1.16–1.44)	1.11 (1.05–1.17)	1.51 (1.39–1.63)
**11th model: 10th model parameters with nonalcoholic Fatty Liver Disease (NAFLD)**				
OR and 95%CI	1	1.29 (1.16–1.45)	1.11 (1.05–1.17)	1.51 (1.40–1.64)
**12th model: 11th model with Obstructive Sleep Apnea (OSA)**				
OR and 95%CI	1	1.30 (1.16–1.45)	1.11 (1.05–1.17)	1.52 (1.40–1.64)
**13th model: 12th model with cardiovascular disease**				
OR and 95%CI	1	1.30 (1.16–1.45)	1.11 (1.05–1.17)	1.52 (1.40–1.64)
**14th model: 13th model parameters with smoking**				
OR and 95%CI	1	1.30 (1.16–1.45)	1.11 (1.05–1.17)	1.50 (1.39–1.63)
**15th model: 14th model parameters and tooth brushing**				
OR and 95%CI	1	1.30 (1.16–1.45)	1.11 (1.05–1.17)	1.50 (1.39–1.63)
**16th model: 15th model parameters with cariogenic nutrition and sugary drinks**				
OR and 95%CI	1	1.18 (1.004–1.39)	1.04 (0.96–1.13)	1.56 (1.39–1.76)

**Table 4:** 16th Model: general linear regression model (GLM) including multicollinearity statistics for the dependent variable carious teeth.

Parameter	B	Standard Error	*p* Value	Exp(B) and 95% Confidence Interval for Exp(B)	Collinearity Statistics	
					Tolerance	VIF
(Intercept)	3.78	0.14	<0.001	44.06 (33.08–58.69)		
Underweight vs. normal weight	0.16	0.08	**0.045**	1.18 (1.004–1.39)	0.838	1.193
Overweight vs. normal weight	0.04	0.04	0.309	1.04 (0.96–1.13)	0.858	1.165
Obesity vs. normal weight	0.45	0.06	**<0.001**	1.56 (1.39–1.76)	0.951	1.051
Age	−0.01	0.004	**0.005**	0.989 (0.981–0.997)	0.288	3.467
Sex: Men vs. women	0.15	0.04	**<0.001**	1.16 (1.07–1.26)	0.922	1.084
Educational level: technicians vs. high school	−0.62	0.08	**<0.001**	0.54 (0.46–0.63)	0.564	1.774
Educational level: academic vs. high school	−0.50	0.07	**<0.001**	0.60 (0.52–0.70)	0.441	2.269
SES: medium vs. low	−1.03	0.08	**<0.001**	0.35 (0.30–0.41)	0.946	1.057
SES: high vs. low	−1.53	0.08	**<0.001**	0.21 (0.18–0.25)	0.937	1.068
Residence location: Urban Jewish vs. Urban non-Jewish	0.34	0.05	**<0.001**	1.41 (1.27–1.57)	0.981	1.020
Residence location: Rural vs. Urban non-Jewish	1.04	0.32	**0.001**	2.82 (1.49–5.33)	0.985	1.015
Birth countries Western Europe vs. Israeli	0.41	0.06	**<0.001**	1.51 (1.33–1.70)	0.983	1.017
Birth countries Eastern Europe vs. Israeli	1.20	0.14	**<0.001**	3.33 (2.49–4.45)	0.980	1.021
Birth countries Asia vs. Israeli	0.03	0.25	0.890	1.03 (0.63–1.69)	0.995	1.005
Birth countries Ethiopia vs. Israeli	0.26	0.13	0.052	1.30 ().98–1.69)	0.986	1.015
Birth countries Africa vs. Israeli	0.02	0.31	0.943	1.02 (0.55–1.88)	0.986	1.015
Birth Countries North America vs. Israeli	−0.63	0.13	**<0.001**	0.53 (0.40–0.69)	0.991	1.010
Birth countries South America vs. Israeli	−0.25	0.19	0.202	0.77 (0.52–1.14)	0.997	1.003
Hypertension	0.08	0.08	0.362	1.08 (0.91–1.29)	0.894	1.118
Diabetes Mellitus	0.22	0.22	0.310	1.25 (0.81–1.93)	0.946	1.057
Hyperlipidemia	−0.14	0.14	0.340	0.86 (0.65–1.16)	0.957	1.045
Nonalcoholic Fatty Liver Disease (NAFLD)	−0.11	0.13	0.412	0.89 (0.67–1.17)	0.907	1.103
Obstructive sleep apnea (OSA)	−0.37	0.23	0.117	0.69 (0.43–1.09)	0.972	1.029
Cardiovascular disease	0.07	0.08	0.409	1.07 (0.90–1.28)	0.920	1.087
Smoking	0.23	0.06	**<0.001**	1.26 (1.11–1.44)	0.750	1.333
Brushing teeth at least once a day	−0.62	0.04	**<0.001**	0.53 (0.49–0.58)	0.793	1.261
Consumption of cariogenic nutrition	0.15	0.04	**0.002**	1.16 (1.06–1.27)	0.589	1.679
Consumption of sugary drinks	0.50	0.04	**<0.001**	1.65 (1.50–1.81)	0.578	1.731

Statistically significant values are in bold. Std.: standard; SES: socio-economic status; vs. versus; VIF: variance inflation factor.

## Data Availability

The data presented in this study are available in article.
